# Cellulose Nanocrystals (CNCs) and Its Modified Form from Durian Rind as Dexamethasone Carrier

**DOI:** 10.3390/polym14235197

**Published:** 2022-11-29

**Authors:** Jindrayani Nyoo Putro, Felycia Edi Soetaredjo, Wenny Irawaty, Sandy Budi Hartono, Shella Permatasari Santoso, Jenni Lie, Maria Yuliana, Widyarani, Hardy Shuwanto, Christian Julius Wijaya, Chintya Gunarto, Nathania Puspitasari, Suryadi Ismadji

**Affiliations:** 1 Department of Chemical Engineering, Widya Mandala Surabaya Catholic University, Kalijudan 37, Surabaya 60114, Indonesia; 2 Collaborative Research Center for Zero Waste and Sustainability, Jl. Kalijudan 37, Surabaya 60114, East Java, Indonesia; 3 Research Center for Environmental and Clean Technology, National Research and Innovation Agency, Samaun Samadikun Science and Technology Center, Sangkuriang, Bandung 40135, Indonesia; 4 Department of Industrial Engineering, Universitas Prima Indonesia, Medan 20117, Indonesia

**Keywords:** cellulose nanocrystals, rarasaponin, sigmoidal

## Abstract

In this study, CNCs were extracted from durian rind. Modification to CNCs with saponin was conducted at 50 °C for one h. CNCs and CNCs-saponin were employed as dexamethasone carriers. Modification to CNCs using saponin did not change the relative crystallinity of CNCs. CNCs’ molecular structure and surface chemistry did not alter significantly after modification. Both nanoparticles have surface charges independently of pH. Dexamethasone-released kinetics were studied at two different pH (7.4 and 5.8). Higuchi, Ritger–Peppas, first-order kinetic and sigmoidal equations were used to represent the released kinetic data. The sigmoidal equation was found to be superior to other models. The CNCs and CNCs-saponin showed burst release at 30 min. The study indicated that cell viability decreased by 30% after modification with saponin.

## 1. Introduction

Various natural polymers, such as cellulose, gelatin, alginate, starch, chitosan, gums, etc., have been studied for their ability and efficiency for drug adsorption and release for potential applications in drug delivery systems. The essential issues in their application as drug carriers are their biodegradability, biocompatibility, and availability in large amounts. One of the available natural polymers which have very potential applications in drug delivery technology is nanocellulose. Due to the high ratio of surface area to pore volume, nanocellulose possesses a higher binding capacity than other natural polymers, thus making it suitable as a drug carrier candidate.

The application of nanocellulose in the advanced drug delivery system is broad; it can be applied either as an excipient or carrier. Currently, nanocelluloses are available in three different forms: cellulose nanocrystals (CNCs), bacterial nanocellulose (BNC), and nanofiber cellulose (NFC) [1]. Among three different forms of nanocelluloses, the CNCs are the most widely studied as drug carriers in advanced drug delivery systems (DDSs). CNCs are obtained from cellulose fibers through different chemical and mechanical processes [2]. The mechanical processes for producing CNCs are expensive; they require specific equipment and are high energy and time-consuming. The chemical treatments, usually using acid as hydrolysis agents, are very efficient for producing CNCs. In the acid hydrolysis process, the disordered amorphous parts in cellulose fibers are destructed while the crystalline parts mostly remain intact.

Currently, the commercial production of CNCs utilizes high-quality biomass materials (wood pulps and cotton) using the acid hydrolysis method. By utilizing high-quality biomasses as raw materials, the cost of raw materials will significantly contribute to the high production cost of the CNCs. Further, global warming and deforestation could make wood pulps unsuitable as raw materials for NCCs production. Various agricultural or low-cost biomass waste types have been explored for their potential utilization as alternative raw materials for CNCs production [2,3,4].

The development of CNCs as nano drug carriers require CNCs, which can limit, control and localize drug release to the targeted site. However, pristine CNCs possess several disadvantages as drug carriers, such as their thermal and pH stability and solubility. The high hydrophilicity of pristine CNCs limits their application as hydrophilic drug carriers [5]. Changing the surface chemistry of CNCs through surface modification processes will make the CNCs more practical for delivering both hydrophilic and hydrophobic drugs.

In this study, CNCs were made from durian rind, which was pretreated with sodium hydroxide solution to remove lignin and hemicellulose content. CNCs were extracted from durian rind cellulose fiber using a sulfuric acid solution. The hydrophobicity of CNCs was enhanced by surface modification using a natural surfactant, rarasaponin, which was extracted from berry soap (*Sapindus rarac DC*). Dexamethasone, a hydrophobic drug, was used as a drug release model. The kinetics and isotherms of the adsorption and release of dexamethasone from CNCs-saponins are also discussed in this paper.

## 2. Materials and Methods

### 2.1. Materials

The durian rind of the golden pillow variety was obtained from a local market in Surabaya, East Java, Indonesia. *Sapindus rarac DC* was obtained from a local market near Kudus City, Central Java, Indonesia. Sodium hydroxide (reagent grade, ≥98%, anhydrous), sulfuric acid (ACS reagent, 95.0–98.0%), methanol (anhydrous, 99.8%), vanillin (≥99%), diosgenin (≥93%), and dexamethasone (powder, ≥98%) were purchased from Sigma-Aldrich, Singapore.

### 2.2. Methods

#### 2.2.1. Pretreatment of Durian Rind

The pretreatment of the durian rind was conducted according to the following procedure. The durian rind was finely sliced and dried in the oven at 105 °C until its moisture content was around 10%. Subsequently, dried durian rind was pulverized until its particle size was found to be a 80/100 mesh. Fifty grams of durian rind powder were added to sodium hydroxide solution (500 mL, 2 N). The mixture was then heated at 80 °C with constant stirring for five hours. After the delignification process, the solid residue was separated from the mixture and repeatedly washed using deionized water. The solid (cellulose) was bleached using hydrogen peroxide (50%) and sodium hydroxide solutions. The mixture was heated at 50 °C for five hours. Subsequently, the bleached cellulose was separated from the solution, repeatedly washed with distilled water, and dried at 105 °C for 24 h.

#### 2.2.2. Extraction of Rarasaponin

The extraction of rarasaponin from the fruit of *Sapindus rarac DC* was conducted using the maceration technique with methanol as the solvent. One hundred grams of dry Sapindus fruit powder was mixed with 500 mL of methanol in a three-neck round bottle. The mixture was heated at 40 °C with constant stirring for 48 h. The solid residue was removed by filtration followed by centrifugation. The filtrate was then transferred to a rotary vacuum evaporator to remove the methanol. After the solvent was removed, the rarasaponin solid extract was collected and stored in a desiccator containing silica gel.

The total saponin in the extract was determined using a colorimetric procedure [6]. This colorimetric method involves the reaction between saponin, sulfuric acid, and vanillin. A UV-vis spectrophotometer at a wavelength of 544 nm was used to measure total saponins; measurements were made using diosgenin as a standard. The saponin content in the extract was 74.5 ± 3.7%.

#### 2.2.3. CNCs Extraction

The extraction of CNCs from the cellulose of the durian rind was conducted using the acid hydrolysis process. The sulfuric acid, with a concentration of 56%, was used as a hydrolysis agent. Ten grams of durian rind cellulose were mixed with 100 mL sulfuric acid. The mixture was heated at 45 °C under constant stirring for 2 h. The solid was removed from the supernatant using centrifugation (9500× *g*, 10 min). Subsequently, the supernatant was placed in dialysis tubing (Spectra Por, MWCO: 10–14 kDa). The free acid in the suspension was removed using reverse osmosis water until the constant pH of the dialysate. The freeze-dried method was used to dry the CNCs.

#### 2.2.4. Modification to CNCs

CNCs modification was carried out by adding 5 g of CNCs and 0.5 g rarasaponin into 100 mL of distilled water and heating at 50 °C for 1 h. During the heating process, the mixture was continuously stirred at 200 RPM. The CNCs-rarasaponin was separated from the solution by centrifugation. The drying of CNCs-rarasaponin was conducted using a freeze dryer.

#### 2.2.5. Characterization of CNCs and CNCs-Rarasaponin

The surface morphology of the samples (CNCs and CNCs-rarasaponin) was characterized using the SEM (Scanning Electron Microscopy, JEOL JSM-6500F field emission SEM) and TEM (HT7700 Transmission Electron Microscopy, Hitachi, Japan) methods. Details of the SEM procedure can be seen elsewhere [7]. The TEM was operated at an accelerating voltage of 80 kV. The crystallinity of CNCs and CNCs-rarasaponin was characterized using X-ray diffraction analysis (XRD, Philips PANalytical X’Pert powder X-ray diffractometer). The diffractograms of the samples were recorded at 30 mA, 40 kV, and a step size of 0.05°/s. Monochromated high-intensity Cu Kα1 was utilized as the radiation source. The crystallinity index (*CrI*) values of raw material, CNCs, and CNCs-rarasaponin were obtained using the following equation

(1) $$CrI=\left(\left(\frac{I_{T}-I_{amo}}{I_{T}}\right)\times 100\right)$$

The symbols *I_T_* and *I_amo_* in Equation (1) represent the intensity of the crystalline and amorph parts of the samples, respectively.

Analysis of the functional groups was conducted using a Shimadzu 8400S FTIR with the KBr method. The wavenumber range of 4000–500/cm was used to record the FTIR spectra of the CNCs and CNCs-rarasaponin. The surface charge of the solid samples was measured using a zeta potential analyzer (NanoBrook ZetaPal). The measurements were conducted at various solution pH and temperatures of 25 °C with 0.01% solid concentration. 0.0001 mol/L NaCl solution was added to the system to maintain the ionic strength in the solution. The particle size of CNCs and CNCs-rarasaponin was also measured using a zeta sizer. The particle size distribution of the samples was calculated using ZetaPals particle sizing software Ver. 5.34.

#### 2.2.6. Drug Loading

In this study, dexamethasone, which is a hydrophobic drug, was used as a model drug. The dexamethasone solution was prepared by dissolving 20 mg of the drug in 10 mL of ethanol. Subsequently, 25 mg of CNCs or CNCs-rarasaponin was added to the dexamethasone solution, and the suspension was sonicated several times. The suspension was shaken in a shaking water bath for 24 h at 30 °C. Dexamethasone-loaded particles were separated by centrifugation and dried under vacuum conditions. A UV-vis spectrophotometer was used to determine the remaining dexamethasone in the solution at 240 nm.

#### 2.2.7. Drug Release Study

A study of dexamethasone release from dexamethasone-loaded CNCs or CNCs-rarasaponin was performed using the membrane dialysis procedure. Dexamethasone-loaded particles with 3 mL of PBS (Phosphate buffer saline) solution (pH 5.8 or 7.4) were added to the dialysis tubing (MWCO = 14,000 Da). The dialysis tubing was placed in a beaker glass containing 47 mL of PBS solution. The drug release experiment was conducted at 37 °C. At a specific time interval, 5 mL of solution was taken from the system. At the same time, an equal volume of fresh PBS solution was added to the system. The concentration of dexamethasone in PBS solution was determined using a UV-vis spectrophotometer.

#### 2.2.8. Cells Viability Study

Cell viability studies were carried out using the MMT assay technique to test the effectiveness and compatibility of CNCs and CNCs-rarasaponin. The 7F2 cell line was used for the MMT cell viability assay. Details of the cell culture can be seen elsewhere [8]. The 7F2 cell line was treated with CNCs, CNCs-rarasaponin, dexamethasone-loaded CNCs, dexamethasone-loaded CNCs-rarasaponin, and no treatment (as the control) to generate a dose–response curve. CNCs, CNCs-rarasaponin, dexamethasone-loaded CNCs, and dexamethasone-loaded CNCs-rarasaponin for the treatment were assessed at 0.25 mg/mL. Before the MMT (3-(4,5-dimethylthiazol-2-yl)-2,5-diphenyltetrazolium bromide) assay, the cells were washed with PBS, and the MMT working solution was added and incubated at 37 °C for four hours. In the cell viability assay, the MMT was converted into formazan [9]. The DMO solution was used to solubilize the formazan crystal.

## 3. Results and Discussions

### 3.1. Characterization of CNCs and CNCs-Rarasaponin

**Figure 1:**
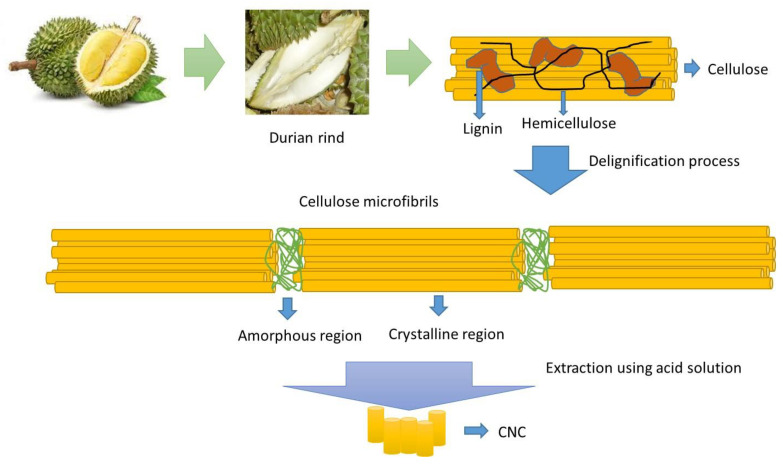
Schematic representation of the delignification and extraction processes of durian rind into CNCs.

The physical and chemical properties of raw materials affect the properties of the CNCs which were obtained from durian rind extraction. The chemical properties of durian rind consist of 35.7 ± 1.6% cellulose, 25.3 ± 1.1% hemicellulose, 28.9 ± 2.1% lignin, and the rest were moisture and ash. After the delignification process, the chemical composition of the durian rind changed to 81.4 ± 3.9% cellulose, 3.4 ± 0.2% hemicellulose, 3.1 ± 0.3% lignin, and the rest were moisture and ash. Most hemicellulose and lignin were successfully removed from the cellulose fibers during the delignification process. The hydrolysis of durian rind cellulose using sulfuric acid solution resulted in the breakdown of β-1,4 glycosidic bonds of glucose monomers in cellulose to obtain CNCs. In the hydrolysis of durian rind cellulose, the hydronium ions (H_3_O^+^) damage the amorphous part while the crystalline part of the cellulose remains intact. H_3_O^+^ efficiently degraded the amorphous region of durian rind cellulose because its structure is less dense than the crystalline parts. A schematic representation of the delignification and extraction processes of durian rind into CNCs is depicted in Figure 1.

**Figure 2:**
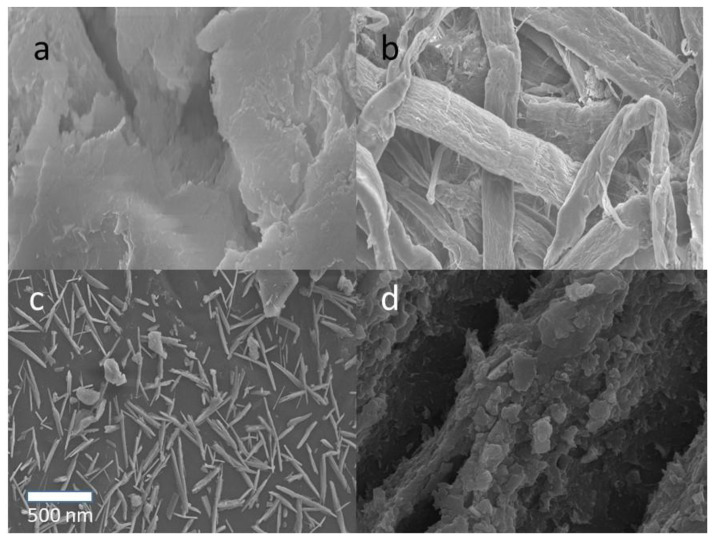
SEM images of (**a**) durian rind, (**b**) durian rind cellulose, (**c**) CNC, and (**d**) CNC-rarasaponin.

**Figure 3:**
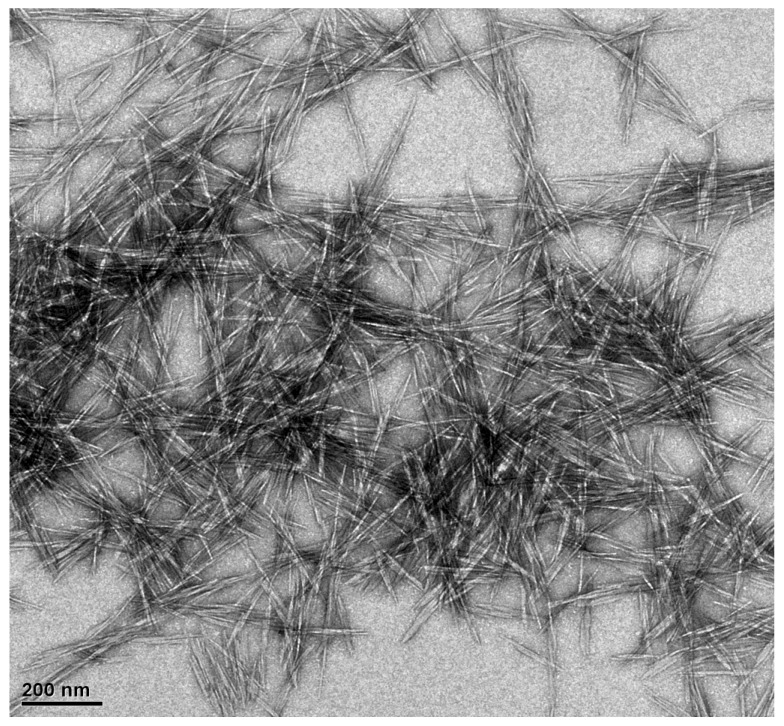
TEM of CNCs from durian rind.

The surface topography of the solid samples was characterized using the SEM method, and the results are shown in Figure 2. After the delignification process, a significant change in the topography of the durian rind surface was seen, as shown in Figure 2a,b. The fiber-like structure of cellulose was observed after removing lignin and hemicellulose from the durian rind. The extraction of the crystalline part from durian rind cellulose produced rod-like nanocrystal (CNCs) with a size around 100–500 nm (Figure 2c). CNCs’ surface topography completely changed after being modified with rarasaponin (Figure 2d). Figure 3 shows the TEM micrograph of the durian rind CNC. Figure 3 reveals that CNCs possesses a needle-like structure with nanoscale dimensions (length 50–250 nm and width 7–10 nm).

**Figure 4:**
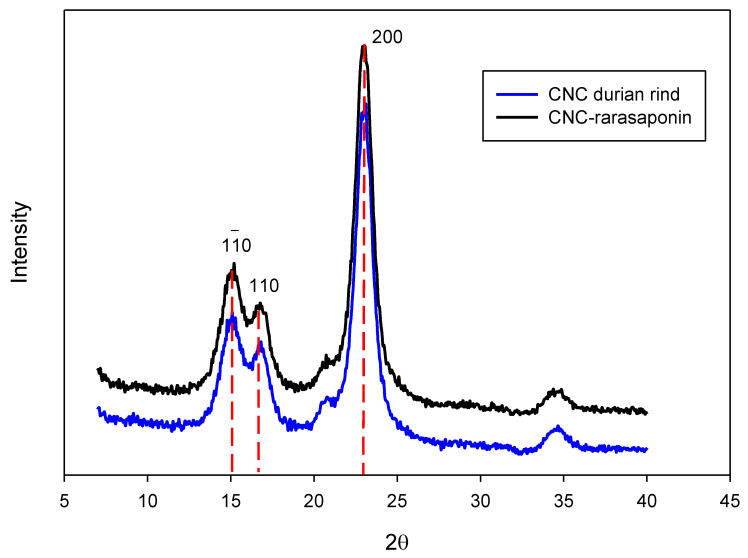
XRD spectra of CNCs durian rind and CNCs-rarasaponin.

XRD analysis of durian skin CNCs and their modified form resulted in a crystalline cellulose pattern (Figure 4). Intense peaks indicate the characteristics of cellulose crystalline at 2θ around 15.1°, 16.6°, and 23.2°. These diffraction peaks correspond to the planes of (1$\bar{1}$0), (110), and (200), respectively, in which the characteristic of cellulose I can be seen. This evidence indicates that the treatment of cellulose using concentrated sulfuric acid did not affect the crystalline part of the native cellulose of durian rind. Modification to CNCs using a natural surfactant (rarasaponin) did not significantly influence the crystallinity index of CNCs (calculated from Equation (1)). The crystallinity index of CNCs originally was 78.8%, which decreased to 77.7% after modification.

**Figure 5:**
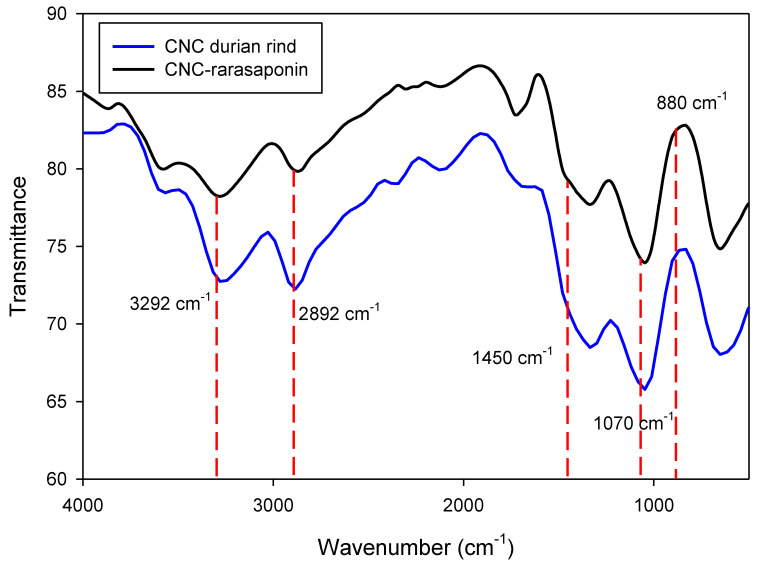
FTIR spectra of durian rind CNCs and CNCs-rarasaponin.

Figure 5 depicts the FTIR spectra of CNCs durian rind and CNCs-rarasaponin. CNCs’ modification using rarasaponin did not significantly change the surface chemistry, as shown in Figure 5. The characteristics of CNCs on the FTIR spectra are shown at wavenumbers of 3292 cm^−1^ (−OH stretching bond), 2892 cm^−1^ (symmetric C−H stretching), 1450 cm^−1^ (−CH_2_−(C_6_) bending), 1070 cm^−1^ (C−O−C (β-glycosidic bond)), and 880 cm^−1^ (C_1_−O−C_4deform_) [10]. These characteristics are similar to the characteristics of cellulose 1.

The surface charges of durian rind CNCs and CNCs-saponin were measured at various pH. Both nanoparticles have surface charges independent of pHs. The surface charge of durian rind CNCs is −22.04 ± 1.45 mV, while the CNCs-rarasaponin has a surface charge of −16.95 ± 1.57 mV. The negative surface charges indicate that both nanoparticles could serve as excellent adsorbents for cationic molecules.

### 3.2. Dexamethasone Loading

Drug-loading experiments were conducted by dissolving a known mass of dexamethasone in ethanol. The maximum uptake of dexamethasone by CNCs was 13.78 mg/g. The concentration of rarasaponin during the modification to CNCs plays a significant role in the uptake of dexamethasone by CNCs-saponin. The rarasaponin concentrations of 5, 10, 15, and 20 mg/L were used for the modification process. Increasing the concentration of rarasaponin from 5 to 15 mg/L increased dexamethasone uptake by CNCs-saponin. However, increased rarasaponin concentration to 20 mg/L decreased dexamethasone uptake by CNCs-saponin. The micelle formation surrounding the CNCs particles reached saturation at a saponin concentration of 15 mg/L. At higher concentrations, some saponin molecules attached to the surface of CNCs-saponin easily dissolve into the ethanol solution. The presence of saponin molecules in an ethanol solution supports the interaction between dexamethasone and ethanol molecules, and the interaction between the surface of CNCs-saponin and dexamethasone is weakened. This phenomenon decreased the uptake of dexamethasone by CNCs-saponin. The maximum dexamethasone uptake by CNCs-rarasaponin was 27.54 mg/g (surfactant concentration during modification was 15 mg/L).

### 3.3. Dexamethasone Released Study

Several well-known models were used to represent the released kinetic of dexamethasone from CNCs and CNCs-saponin. These are the Higuchi [11], Ritger Peppas [12], first-order kinetic [13], and sigmoidal models [8]. The Higuchi equation is possibly the first model that quantifies drug release from thin films. Due to its simplicity [14], the Higuchi equation is widely used to correlate the kinetic of drugs released from various solid systems [8,15,16,17,18,19,20]. Th Higuchi equation has the following for:

(2) $$\frac{n_{t}}{n_{max}}=k_{H}\sqrt{t}$$

where *n_t_* is the cumulative amount of the drug released at time *t*, while *n_max_* is the maximum amount of drug that can be released at an infinite time. The parameter *k_H_* represents the Higuchi constant.

Several methods have been developed to describe the behavior of non-Fickian dispersion in the solvent uptake by the polymers. In those approaches, the sharp change in solvent diffusivity across the swelling substances is usually represented by a time- or position-dependent diffusion coefficient [21]. Time dependency on the swelling behavior of the polymers in the simplified exponential form can be written as [12]:

(3) $$\frac{n_{t}}{n_{max}}=k_{RP}t^{n}$$

where *k_RP_* and *n* are the Ritger–Peppas parameters. The parameter *n* could represent the drug release mechanism. For the thin film, if the value of exponent *n is* equal to 0.5, the drug release mechanism is Fickian diffusion. Supposing the value of *n* is 0.5< *n* <1.0, the mechanism would be anomalous transport. When *n* equals 1, the drug release mechanism is case-II transport. For a polymeric-controlled delivery system with cylinder geometry, the Fickian diffusion mechanism occurs when the value of *n* is 0.45. The anomalous transport occurs when 0.45 < *n* < 0.89. The case-II transport mechanism is represented by the *n* value of 0.89. For sphere geometric, the Fickian diffusion mechanism is represented by the *n* value of 0.43, the anomalous transport by 0.43 < *n* < 0.85, and the case-II transport mechanism by the *n* value of 0.85 [14].

Lin et al. [13] proposed a released kinetic model based on mass transfer and interaction between ionic solution and the drug. Their model can be written as

(4) $$\frac{n_{t}}{n_{max}}=\left(1-exp\left(-K_{L}a.t\right)\right)$$

where *K_L_a* is the volumetric mass transfer coefficient. Equation (4) is similar to the first-order kinetic model. The main feature of Lin’s model is that the drug carrier is non-erodible and non-swellable.

The sigmoidal-released kinetic model is an empirical equation proposed by Putro et al. [8] to represent the released kinetic of paclitaxel from surfactants-modified polysaccharides nanoparticles. The sigmoidal-released kinetic model has a mathematical form as follows

(5) $$\frac{n_{t}}{n_{max}}=\frac{R_{max}}{1+exp\left(-\left(t-t_{50}\right)/ks\right)}$$

Parameter *R_max_* represents the theoretical maximum release percentage. The parameter *ks* is the release rate, while *t_50_* is the time taken to achieve 50% of the maximum release.

**Figure 6:**
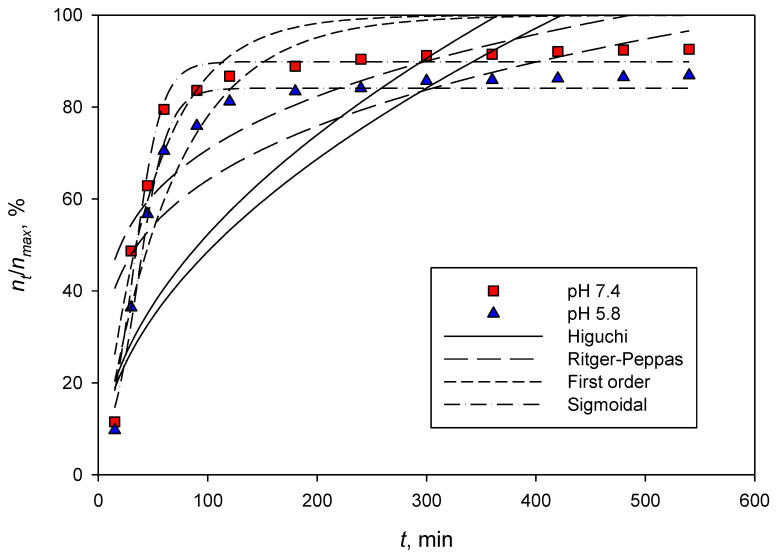
Dexamethasone released from CNCs-rarasaponin at 37 °C.

**Figure 7:**
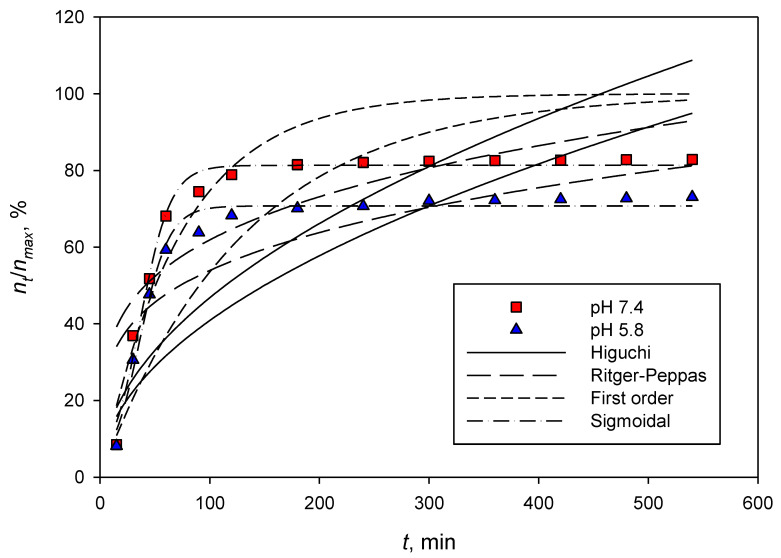
Dexamethasone released from durian rind CNCs at 37 °C.

Figure 6 and Figure 7 depict the dexamethasone released from CNCs-rarasaponin and Durian rind CNCs. The experimental data were obtained at 37 °C and two different pHs, 5.8 and 7.4. The fitting parameters of the Higuchi, Ritger–Peppas, first-order, and sigmoidal models are summarized in Table 1. Visually, the Higuchi, Ritger–Peppas, and first-order models fail to represent the dexamethasone release from the CNCs and CNCs-rarasaponin. This evidence was also supported by low values of *R^2^* of Higuchi, Ritger–Peppas, and first-order models (Table 1).

Higuchi equation was developed to represent the release of drugs from a thin ointment film into the skin. Many assumptions were used to develop this model in order to obtain a simple equation. Higuchi’s model employed a steady-state approach; this approach is only valid for the systems with an excess amount of drug at the initial condition. This condition was not met in this study; therefore, the Higuchi equation fails to represent the release of dexamethasone from CNCs and CNCs-saponin. Similar to the Higuchi equation, Ritger–Peppas failed to describe the release kinetics of dexamethasone from CNCs and CNCs-saponin. The parameter *n* is much smaller than 0.5, and *R^2^* is in the range of 0.6799 to 0.7799. Ritger–Peppas was developed for swelling polymers, which are completely different from our system. Like the Higuchi and Ritger–Peppas models, the first-order model cannot describe the experimental data well.

The sigmoidal model could represent the experimental data well, as shown in Figure 6 and Figure 7. The maximum or theoretical drug release can also be predicted well with parameter Rmax, and the values are consistent with the experimental data in Figure 6 and Figure 7. The values of parameter *T_50_* are also consistent with the experimental data. Even the sigmoidal model is an empirical equation; this model is flexible since it contains one more parameter than other models used in this study.

### 3.4. Biocompability of CNCs and CNCs-Rarasaponin

**Figure 8:**
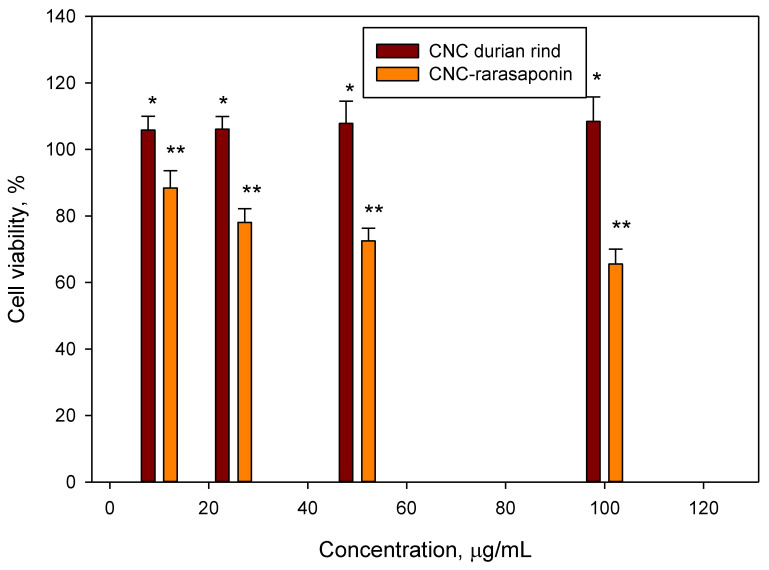
Cell viability of CNCs and CNCs-rarasaponin. Data represent the mean ± SD (*n* = 3), * indicates *p* < 0.05, and ** indicates *p* < 0.01 (statistically significant difference).

The biocompatibility of CNCs and CNCs-rarasaponin was tested against a 7F2 cell line, and the results are given in Figure 8. As seen in Figure 8, CNCs are not toxic to osteoblast cells. There were high cell viabilities in CNCs, possibly due to slow diffusion from the surface of CNCs to the cell surface. After modification with rarasaponin, cell proliferation decreased with the increase in cell concentration. At 100 μg/mL suspension, the cell viability decreased by 30%. Naturally, saponin is cytotoxic toward specific tumor cells; to a normal cell, saponin also has nonspecific activity. Slight apoptotic changes in normal cells can be induced by saponin (in this case, rarasaponin). Therefore, direct contact between saponin and cells will promote cell lysis immediately [22,23].

## 4. Conclusions

The extraction of CNCs from durian rind was conducted using concentrated sulphuric acid and produced a rod-like nanocrystal (CNCs) size of around 100–500 nm. CNCs’ surface topography completely changed after modification with rarasaponin. Both particles, CNCs and CNCs-saponin, have negative surface charges. The sigmoidal model gave the best result among several well-known models used to represent the released kinetic of dexamethasone from CNCs and CNCs-saponin. High cell viability in CNCs was observed. The cell viability decreased by 30% after modification with saponin.

## Figures and Tables

**Table 1:** Parameters of several released kinetic models.

pH	Drug Carrier	Parameters			
		Higuchi	Ritger–Peppas	First-order	Sigmoidal
7.4	CNCs-rarasaponin	*K_H_* = 5.2307 *R^2^* = 0.0026	*K_RP_* = 25.90 *n* = 0.2184 *R^2^* = 0.6799	*K_L_a* = 0.0202 *R^2^* = 0.8822	*R_max_* = 89.88 *T_50_* = 32.18 *k_s_* = 12.59 *R^2^* = 0.9739
	Durian rind CNCs	*K_H_* = 4.6792 *R^2^* = 0.2107	*K_RP_* = 20.42 *n* = 0.2409 *R^2^* = 0.6968	*K_L_a* = 0.0137 *R^2^* = 0.6848	*R_max_* = 81.36 *T_50_* = 36.41 *k_s_* = 13.92 *R^2^* = 0.9833
5.8	CNCs-rarasaponin	*K_H_* = 4.8576 *R^2^* = 0.2298	*K_RP_* = 20.99 *n* = 0.2426 *R^2^* = 0.7117	*K_L_a* = 0.0151 *R^2^* = 0.7833	*R_max_* = 84.13 *T_50_* = 35.92 *k_s_* = 13.39 *R^2^* = 0.9827
	Durian rind CNCs	*K_H_* = 4.0853 *R^2^* = 0.2298	*K_RP_* = 17.65 *n* = 0.2126 *R^2^* = 0.7799	*K_L_a* = 0.0077 *R^2^* = 0.1644	*R_max_* = 70.76 *T_50_* = 35.91 *k_s_* = 13.39 *R^2^* = 0.9827

## Data Availability

All the data have been given in the manuscript.
